# Modifiable risk factors for typhoid intestinal perforations during a large outbreak of typhoid fever, Kampala Uganda, 2015

**DOI:** 10.1186/s12879-017-2720-2

**Published:** 2017-09-25

**Authors:** Lilian Bulage, Ben Masiira, Alex R. Ario, Joseph K.B Matovu, Peter Nsubuga, Frank Kaharuza, Victoria Nankabirwa, Janell Routh, Bao-Ping Zhu

**Affiliations:** 1 Uganda Public Health Fellowship Program – Field Epidemiology Track, Ministry of Health – Makerere University School of Public Health, P.O. Box 7072, Kampala, Uganda; 2 African Field Epidemiology Network, Kampala, Uganda; 3 Department of Epidemiology and Biostatistics, School of Public Health, College of Health Sciences, Makerere University, Kampala, Uganda; 4 National Center for Immunizable and Respiratory Diseases ,Centers for Disease Control and Prevention, Atlanta, USA

**Keywords:** Typhoid fever outbreak, Intestinal perforations, Uganda

## Abstract

**Background:**

Between January and June, 2015, a large typhoid fever outbreak occurred in Kampala, Uganda, with 10,230 suspected cases. During the outbreak, area surgeons reported a surge in cases of typhoid intestinal perforation (TIP), a complication of typhoid fever. We conducted an investigation to characterize TIP cases and identify modifiable risk factors for TIP.

**Methods:**

We defined a TIP case as a physician-diagnosed typhoid patient with non-traumatic terminal ileum perforation. We identified cases by reviewing medical records at all five major hospitals in Kampala from 2013 to 2015. In a matched case-control study, we compared potential risk factors among TIP cases and controls; controls were typhoid patients diagnosed by TUBEX TF, culture, or physician but without TIP, identified from the outbreak line-list and matched to cases by age, sex and residence. Cases and controls were interviewed using a standard questionnaire from 1st -23rd December 2015. We used conditional logistic regression to assess risk factors for TIP and control for confounding.

**Results:**

Of the 88 TIP cases identified during 2013–2015, 77% (68/88) occurred between January and June, 2015; TIPs sharply increased in January and peaked in March, coincident with the typhoid outbreak. The estimated risk of TIP was 6.6 per 1000 suspected typhoid infections (68/10,230). The case-fatality rate was 10% (7/68). Cases sought care later than controls; Compared with 29% (13/45) of TIP cases and 63% (86/137) of controls who sought treatment within 3 days of onset, 42% (19/45) of cases and 32% (44/137) of controls sought treatment 4–9 days after illness onset (OR_adj_ = 2.2, 95%CI = 0.83–5.8), while 29% (13/45) of cases and 5.1% (7/137) of controls sought treatment ≥10 days after onset (OR_adj_ = 11, 95%CI = 1.9–61). 68% (96/141) of cases and 23% (23/100) of controls had got treatment before being treated at the treatment centre (OR_adj_ = 9.0, 95%CI = 1.1–78).

**Conclusion:**

Delay in seeking treatment increased the risk of TIPs. For future outbreaks, we recommended aggressive community case-finding, and informational campaigns in affected communities and among local healthcare providers to increase awareness of the need for early and appropriate treatment.

## Background

Typhoid fever is a bacterial infection caused by *Salmonella enterica* serotype Typhi and characterized by insidious onset of sustained fever, marked headache, abdominal pain, constipation, and diarrhoea. Transmission is through ingestion of water and food contaminated by faeces of infected persons or carriers [1]. Globally, an estimated 21 million cases and 222,000 deaths due to typhoid fever occur annually [2]. In Africa, the estimated annual incidence ranges from 13 to 845 per 100,000 [3–5].

Most typhoid patients with prompt and appropriate management have excellent prognosis, with low estimated case-fatality rate [6]. However, some typhoid patients develop life-threatening complications such as intestinal perforation (IP) and consquent peritonitis. Chemical peritonitis due to leakage of the gastric acid into the peritoneal cavity may also occur [1]. The case fatality rate in typhoid patients who develop an IP ranges from 6 to 40% [7–11]. Surgical intervention within the first 24 hours of perforation, aggressive resuscitation and treatment with appropriate antibiotics are critical in improving survival of patients with intestinal perforation [12].

Typhoid fever is confirmed by isolation of *S.*Typhi in bone marrow or blood in the early stages of the disease [1, 13]. However, culture of clinical specimens is resource-intensive and is frequently unavailable in resource-limited settings. Therefore, many countries rely on rapid diagnostic tests such as the Widal test [14] or TUBEX-TF [15] for laboratory diagnosis of typhoid; yet these tests do not have adequate sensitivity and specificity for the diagnosis of individual patients, which in turn hinders the accuracy of surveillance data [10–12]. The problem is further complicated in malaria endemic areas of sub-Saharan Africa because typhoid and malaria have similar presentations [1].

Between January and June, 2015, a large typhoid outbreak occurred in Kampala, causing 10,230 suspected infections [16]. Area surgeons noticed an increase in the number of IPs during the 2015 outbreak period. We conducted an investigation to ascertain whether the number of typhoid intestinal perforations (TIPs) had actually increased, and to identify modifiable risk factors associated with TIP during this outbreak in order to inform prevention and control measures.

## Methods

### Case definition and case finding

We defined a case of non-traumatic IP as an onset of physician-diagnosed perforation in any part of the intestine of a patient who did not have a recent trauma or injury that could explain the perforation. A physician diagnosed perforation was defined as a non-traumatic hole in any part of the wall of the gastro-intestinal tract which lines the stomach, small intestine or large bowel. A case of TIP was onset of non-traumatic IP in the terminal ileum of a physician-diagnosed typhoid patient. A case of other IP was onset of non-traumatic IP that occurred in regions other than the terminal ileum or otherwise did not meet the definition of a TIP.

Using a standardized data abstraction form, we reviewed theatre registers and case files kept at five major hospitals (described below) in Kampala City to identify all IPs, TIPs, and other IPs that occurred between January 2013 and December 2015.

### Study site

Kampala is the capital city of Uganda situated in the central region of Uganda and had an estimated population of 1,516,210 based on the 2014 census [17]. The city is made up of five administrative divisions: Central, Nakawa, Makindye, Rubaga and Kawempe. We conducted the study among Kampala city residents who had been admitted in the hospitals mentioned below for TIP and patients with a diagnosis of typhoid fever without perforation. The study was conducted at five major hospitals within Kampala: Mulago National Referral Hospital, Naguru Regional Referral Hospital, Nsambya Hospital, Mengo Hospital, and Rubaga Hospital. Mulago is a public, national referral hospital which admits patients from Kampala and all over the country for free treatment. Naguru Hospital is a public regional referral hospital which admits patients from Kampala and the surrounding districts for free treatment. Nsambya, Mengo, and Rubaga hospitals are private not-for-profit hospitals that serve Kampala and surrounding districts. We selected those hospitals because patients with IPs were most likely to be referred to these high-level facilities, since the lower level health centres may not have had the capacity to handle such cases. The outbreak investigations indicated that most of the typhoid fever case-persons were of low socio-economic status [16]; therefore they are not expected to seek healthcare services in expensive private hospitals in Kampala.

### Case-control study

We recruited all identified TIP case-patients who were alive at the time of our investigation to participate in our case-control study from 1st-23rd December 2015. Controls were residents of Kampala City line-listed during the outbreak, who were diagnosed with typhoid fever by either TUBEX-TF or blood culture confirmation, aged ≤65 years, and did not have an IP. TUBEX-TF is a rapid in vitro diagnostic test for diagnosis of acute typhoid fever [15]. For each case, we recruited three controls, randomly selected from a line list generated during the 2015 typhoid outbreak, individually matched by sex, age, and division of residence. In determining the case-to-control ratio, we estimated that approximately 50 cases could be successfully recruited and interviewed. Assuming the proportion of exposure [clinical information (pre-existing medical condition(s) such as TB, HIV, Diabetes and Cancer; duration of illness; duration of symptoms in days; duration before seeking treatment); where the first treatment was sought (i.e., self-medication, drug shop, private clinic, or hospital/health centre); number of clinics visited; and knowledge about typhoid before they contracted the disease (i.e., whether they had heard of a disease called “typhoid” before; whether they had heard of a typhoid outbreak in Kampala during the first half of 2015] in the control group was 30%, to detect an odds ratio (OR) of ≥2.5 with 95% confidence and a power of 80%, one would need approximately 150 controls, for a case-to-control ratio of 1:3. The estimated OR of ≥2.5 was based on a study conducted in Turkey, which found ORs ranging from 1.2 to 5.0 for the different risk factors identified [18].

We used a structured questionnaire to interview the case- and control-persons in person whenever they were available, or otherwise by telephone. For study participants aged <18 years, we interviewed their parents or care-givers instead.

### Study variables

We collected information on the following variables: socio-demographic factors (age, sex, level of education, marital status and place of residence); clinical information (pre-existing medical condition(s) such as TB, HIV, Diabetes and Cancer; duration of illness; duration of symptoms in days; duration before seeking treatment (i.e., the number of days from the onset of symptoms to the time of receiving first treatment from any health facility); where the first treatment was sought (i.e., self-medication, drug shop, private clinic, or hospital/health centre); number of clinics visited; and knowledge about typhoid before they contracted the disease (i.e., whether they had heard of a disease called “typhoid” before; whether they had heard of a typhoid outbreak in Kampala during the first half of 2015).

### Data analysis

We evaluated the association between each individual risk factor and TIP using conditional logistic regression to account for the matched study design, and calculated the crude odds ratio (OR_crude_) and its associated 95% confidence intervals (CI) for each risk factor. To adjust for confounding, we used multivariable conditional logistic regression by including co-variates in the model, and calculated the adjusted odds ratio (OR_adj_) and their associated 95% CI [19]. Co-variates that were significant at the *p* < 0.05 level were retained in the multivariable conditional logistic regression model.

## Results

### Trends of TIPs and other IPs, January 2013–December 2015

**Fig. 1:**
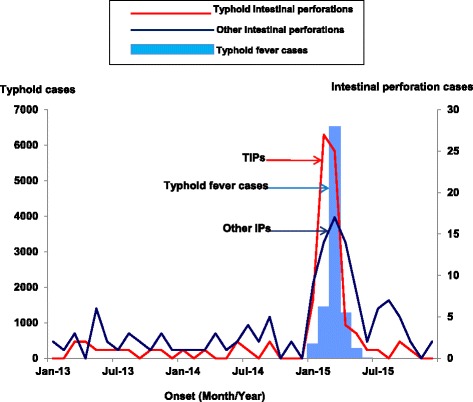
Typhoid cases, typhoid intestinal perforations, and other intestinal perforations, January 2013–December 2015, Kampala. The red coloured line graph represents typhoid intestinal perforations that were recorded during the 2015 typhoid fever outbreak in Kampala. The dark blue line graph represents other intestinal perforations that were recorded during the 2015 typhoid fever outbreak in Kampala. The light blue histogram (epi-curve) represents typhoid fever cases that were recorded during the 2015 typhoid fever outbreak in Kampala

Between January 2013 and December 2014, the number of TIPs ranged between 0 and 2 per month and remained below other IPs (Fig. 1). However, the number of TIPs dramatically increased during the outbreak period (January to June) in 2015, with a peak observed in February and March. In total, 68 cases of IPs were identified during the outbreak period. The outbreak resulted in an estimated 10,230 suspected typhoid infections [16]; therefore, the estimated minimum risk of developing TIP was 6.6 (68/10,230) per 1000 suspected infections. Of the 68 TIP case-persons identified during the outbreak period, 7 died (according to patient files and surgical log books), yielding a case-fatality rate of 10%.

Between January 2013 and December 2014, cases of other IPs remained stable, ranging from 0 to 6 cases per month. As with the cases of TIP, the number of other IPs also sharply increased in January, with a peak observed in March 2015 (Fig. 1). In total, 70 cases of other IPs were identified during the outbreak period.

### Risk factors for TIP, January–June 2015

Of the 58 TIP case-persons whose medical records were available, 50 (86%) agreed to participate in the case-control study. The majority (86%) of TIP case-persons were males. The mean number of days from the development of symptoms to hospitalization was 11 days (range: 1–42 days). The majority of TIP case-persons (59%) were hospitalized after 7 days of illness onset. Complications after surgery occurred in 40% of TIP case-persons, with the commonest complication (70%) being wound infection (Table 1). No cases of intestinal tuberculosis, HIV, diabetes, and cancer were reported in the study population between January to June 2015.

**Table 1:** Socio-demographic and clinical characteristics of cases and controls, Kampala, Uganda, January–June 2015

Characteristic	% TIP cases (*N* = 50)	% Controls^a^ (*N* = 145)	*P*-Value
Gender			
Male	86	87	0.87
Female	14	13	
Mean age(±SD)	28(±8.4)	28(9.0)	0.96
Education level			
None	6	3	0.57
Primary	28	21	
Secondary	47	55	
Tertiary	19	21	
Number of clinics visited before			
0	32	26	0.51
1	50	57	
2	13	14	
3	3	3	
4	3	0	
Time to admission (Days)			
< 7	40	–	
7–13	30	–	
≥ 14	30	–	
Post-surgery complications			
None	60	–	
Burst abdomen	2	–	
Fistula	2	–	
Repeat surgeries	4	–	
Wound gaping	4	–	
Wound infection	28	–	

*TIP* Typhoid intestinal perforation

^a^Controls were typhoid fever cases without intestinal perforation matched by sex, age, and division of residence to cases

Of the 50 TIP cases, 45 had complete data on risk factors and were used for the univariate and conditional logistic regression analysis. Cases and controls did not differ significantly in gender, mean age, education level, and the number of clinics visited before diagnoisis of TIP (Table 1). When comparing the cases and controls by individual risk factors using conditional logistic regression, risk of TIP increased with the length of time before seeking treatment: Seeking treatment ≥10 days after illness onset (OR_crude_ = 13, 95%CI = 3.8–43) and 4–9 days after illness onset (OR_crude_ = 3.0,95%CI = 1.8–5.6) were associated with significantly increased risk for TIP, compared with seeking care within 3 days of illness onset. Similarly, self-medication before seeking formal treatment (OR_crude_ = 2.9,95%CI = 1.4–6.2), having not heard about the typhoid outbreak in Kampala (OR_crude_ = 4.4,95%CI = 1.9–10.3), having got treatment before being treated at the typhoid treatment center (OR_crude_ = 7.0,95%CI = 1.5–31), and having not heard about typhoid fever at all (OR_crude_ = 2.5,95%CI = 1.0–5.9) were also significantly associated with TIP (Table 2).

**Table 2:** Modifiable Risk factors for Typhoid Intestinal Perforations during an outbreak of typhoid fever in Kampala, Uganda, January–June 2015

Variable	% of TIP cases (*N* = 45)	% of controls^a^ (*N* = 137)	OR_crude_ (95% CI)	OR_adj_ (95% CI)
Days before seeking treatment				
0–3	29	63	ref	
4–9	42	32	3.0(1.8–5.7)	2.2(0.83–5.8)
10+	29	5.1	13(3.8–43)	11(1.9–61)
Heard of typhoid fever before				
Yes	64	82	ref	
No	36	18	2.5(1.0–5.9)	NS
Heard of typhoid outbreak in Kampala				
Yes	41	75	ref	
No	59	25	4.4(1.9–10)	5.2(1.8–15)
Got treatment before^b^				
No	45	77	ref	
Yes	96	23	7.0(1.5–31)	9.0(1.1–78)

*TIP* Typhoid intestinal perforation, *OR*_*crude*_ Crude odds ratio from conditional logistic regression (for the matched design), *OR*_*adj*_ Adjusted odds ratio using multivariable conditional logistic regression, *CI* confidence interval, *NS* not statistically significant (therefore not included in the conditional logistic regression model)

^a^Controls were typhoid fever cases without intestinal perforation

^b^Treatment received before being admitted for TIP or being treated at a typhoid treatment centre. This treatment was mainly received from drug shops and private clinics

When we used conditional logistic regression to control for confounding, seeking care 10+ days after onset of symptoms remained significantly associated with TIP (OR_adj_ = 11, 95% CI = 1.9–61), as was having received treatment before being treated at the typhoid treatment center (OR_adj_ = 9.0, 95%CI = 1.1–78), and being uninformed of the current typhoid outbreak (OR_adj_ = 5.2, 95% CI = 1.8–15) (Table 2). The associations between other risk factors and TIP became statistically non-significant in the logistic regression analysis (Table 2).

## Discussion

Our investigation uncovered an outbreak of IPs (both TIPs and other IPs) during January to June 2015, coinciding with the large typhoid outbreak in Kampala in 2015. Development of TIP was significantly associated with the length of time from onset of symptoms to seeking treatment, having got treatment before being treated at the typhoid treatment center, and with not having heard about the typhoid outbreak in Kampala City.

In recent years, severe typhoid outbreaks have been reported in African countries, including Blantyre Malawi [20], Uganda [21], and Zimbabwe [22], resulting in a large number of infections and deaths, with TIP as a leading cause of death.

TIP usually occurs in the third week of illness, but can occur as early as the second week of illness, especially in developing countries, for reasons that are not well-understood [12]. Development of severe typhoid infection including TIP has been linked to resistant *S.* Typhi strains and delay in initiation of appropriate antimicrobial therapy [1, 23, 24]. Therefore, early diagnosis and prompt treatment are the keys for preventing TIP. Our investigation has provided cooroborating evidence by showing that delay in seeking medical care was associated with TIP.

Surveillance for typhoid fever in low income countries has always been a challenge because positive identification of a *S.* Typhi infection relies on blood or bone-marrow culture [1], which is cost-prohibitive in most low-income countries. In Uganda, the laboratory diagnosis of typhoid fever is largely based on the Widal Test [25], which has little diagnostic value; consequently, the resulting typhoid surveillance data are unreliable for outbreak detection. Our findings that a sharp increase in TIP (and in other IP) coincided with the typhoid fever outbreak showed that it might be possible to use the increase in intestinal perforations as a sentinel event to complement typhoid surveillance data for outbreak detection in resource-limited countries.

Even though TIP is known to normally occur in the terminal ileum area of the intestine, our investigation showed that “other IPs” also had a notable increase during the 2015 outbreak period. This observation implies that these “other IPs” may have been a result of *S.* Typhi infection during the outbreak, consistent with published literature showing that IPs in other sites of the gut (such as upper ileum, caecum and jejunum) can also be caused by *S.* Typhi infection [26, 27]. Therefore, the burden due to IP during the Kampala typhoid outbreak likely was much larger than the 68 TIP cases we identified.

## Limitations

Our study had several limitations. Since this was a hospital based study, the reported TIPs are minimum incidence estimates and hence the population incidence of TIPs was not estimated. Some of the controls were only TUBEX-positive rather than culture-confirmed. Even though TUBEX-TF has been shown to be a better test than the Widal Test [1], the diagnostic reliability is still limited. Therefore, some of the controls might have been misdiagnosed as typhoid fever when in fact they were not. There might have been recall bias since the study was conducted 6 months after the typhoid outbreak had ended. Patients with TIP might have been more likely to remember their symptoms and risk factors compared to those without TIP. In-addition, our patients could hardly remember the details of the treatments they received which could have been key in differentiating the medications taken and subsequent understanding of the outcomes. Given the study design, it is likely that we under-estimated the proportion of TIP deaths associated with the 2015 typhoid fever outbreak in Kampala. It is possible that some of the patients died in the communities without accessing health care. While as general information on the overall case fatality rate, regardless of TIP would be useful in understanding the contribution of TIP to overall mortality during the outbreak, data on the general mortality due to the outbreak was not collected [16]. Our case-control study was only able to collect complete data from 45 (66%) of the 68 TIP case-persons, potentially resulting in selection bias, which could have biased the risk estimates in any direction.

## Conclusions and recommendations

In conclusion, delay in seeking treatment, having got treatment before being treated at the typhoid treatment center, and being uninformed of the ongoing outbreak were associated with an increased risk of TIP during the 2015 typhoid fever outbreak in Kampala. We recommend aggressive community case-finding for early and appropriate treatment, and strengthening health education about typhoid fever and TIPs and their prevention during future typhoid outbreaks.

## Abbreviations

AIDS: Acquired immunodeficiency syndrome
CDC: Centers for Disease Control and Prevention
CI: Confidence interval
HIV: Human immunodeficiency virus,
IP: Intestinal perforations
OR: Odds ratio
OR_adj_: Adjusted odds ratio
OR_crude_: Crude odds ratio
PEPFAR: President’s emergency plan for AIDS relief
TB: Tuberculosis
TIP: Typhoid intestinal perforations

## References

[1] Heymann DL (2014). Control of communicable diseases manual.

[2] Crump JA, Luby SP, Mintz ED (2004). The global burden of typhoid fever. Bulletin of the World Health Organisation.

[3] Crump JA, Luby SP, Mintz ED (2004). The global burden of typhoid fever. Bull World Health Organ.

[4] Crump JA (2008). Part I. Analysis of data gaps pertaining to salmonella enterica serotype Typhi infections in low and medium human development index countries, 1984-2005. Epidemiol Infect.

[5] Crump JA (2003). Estimating the incidence of typhoid fever and other febrile illnesses in developing countries. Emerg Infect Dis.

[6] Bhutta ZA (2006). Current concepts in the diagnosis and treatment of typhoid fever. BMJ.

[7] Butler T (1985). Typhoid fever complicated by intestinal perforation: a persisting fatal disease requiring surgical management. Rev Infect Dis.

[8] Eustache JM, Kreis DJ (1983). Typhoid perforation of the intestine. Arch Surg.

[9] Oheneh-Yeboah M (2007). Postoperative complications after surgery for typhoid ileal perforation in adults in Kumasi. West Afr J Med.

[10] Parry CM (2002). Typhoid fever. N Engl J Med.

[11] van Basten JP, Stockenbrugger R (1994). Typhoid perforation. A review of the literature since 1960. Trop Geogr Med.

[12] WHO (2003). Background document: the diagnosis, treatment and prevention of typhoid fever.

[13] Wain J, Hosoglu S (2008). The laboratory diagnosis of enteric fever. J Infect Dev Ctries.

[14] Senewiratne B, Senewiratne K (1977). Reassessment of the Widal test in the diagnosis of typhoid. Gastroenterology.

[15] Biotech. TUBEX TF Rapid typhoid detection. Available from: http://www.idlbiotech.com/Documents/Broschyrer,%20leaflet/IDL_TUBEX_201511.pdf. Accessed 15 Jan 2016.

[16] Kabwama SN, Bulage L, Nsubuga F, Pande G, Oguttu DW, Mafigiri R, et al. A large and persistent outbreak of typhoid fever caused by consuming contaminated water and street-vended beverages: Kampala, Uganda, January – June 2015. BMC Public Health. 2017;17(1):23. 10.1186/s12889-016-4002-0 PMC5216563 28056940

[17] Uganda Bureau of Statistics, National Population and Housing Census 2014*.* Provisional Results, November 2014.

[18] Hosoglu S, Aldemir M, Akalin S. Risk factors for enteric perforation in patients with typhoid fever. Am J Epidemiol. 2004;160(1):46–50. 10.1093/aje/kwh172 15229116

[19] Gregg M, Field epidemiology. 3rd ed. Oxford; New York; Oxford University Press; 2008.

[20] Lutterloh E (2012). Multidrug-resistant typhoid fever with neurologic findings on the Malawi-Mozambique border. Clin Infect Dis.

[21] Neil KP (2012). A large outbreak of typhoid fever associated with a high rate of intestinal perforation in Kasese District, Uganda, 2008-2009. Clin Infect Dis.

[22] CDC (2012). Notes from the field: salmonella Typhi infections associated with contaminated water--Zimbabwe, October 2011-may 2012. MMWR Morb Mortal Wkly Rep.

[23] Ahmed HN (2006). Typhoid perforation still a common problem: situation in Pakistan in comparison to other countries of low human development. JPMA. J Pak Med Assoc.

[24] Parry CM (2014). Risk factors for the development of severe typhoid fever in Vietnam. BMC Infect Dis.

[25] Olopoenia LA, King AL (2000). Widal agglutination test− 100 years later: still plagued by controversy. Postgrad Med J.

[26] Ekenze SO, Ikefuna AN (2008). Typhoid intestinal perforation under 5 years of age. Ann Trop Paediatr.

[27] van der Werf TS, Cameron FS (1990). Typhoid perforations of the ileum. A review of 59 cases, seen at Agogo hospital, Ghana, between 1982 and 1987. Trop Geogr Med.

